# Single-cell and machine learning integration reveals ferroptosis-driven immune landscapes for melanoma stratification

**DOI:** 10.3389/fimmu.2025.1624691

**Published:** 2025-08-01

**Authors:** Lei Wang, Xueying Jin, Yuchen Wu, Runing Qiu, Jianfang Wang

**Affiliations:** ^1^ Department of Oncology, Shaoxing People’s Hospital, The First Affiliated Hospital of Shaoxing University, Shaoxing, Zhejiang, China; ^2^ Laboratory of Cancer Biology, Key Lab of Biotherapy in Zhejiang Province, Cancer Center of Zhejiang University, Sir Run Run Shaw Hospital, School of Medicine, Zhejiang University, Hangzhou, Zhejiang, China

**Keywords:** ferroptosis, melanoma, immune microenvironment, single-cell RNA sequencing, machine learning

## Abstract

**Background:**

Ferroptosis, a regulated form of cell death, has emerged as a critical modulator of melanoma's tumor progression and immune evasion. However, its integration with the tumor immune microenvironment (TME) and clinical prognostication remains underexplored. This study aims to construct a multi-omics framework combining ferroptosis-related signatures, immune infiltration patterns, and machine-learning approaches to stratify melanoma patients and guide therapeutic decision-making.

**Methods:**

We developed a multi-omics framework integrating bulk transcriptomics (TCGA/GEO), single-cell RNA sequencing, and machine learning to decode melanoma's ferroptosis-immune axis. Ferroptosis-immune subtypes were identified through consensus clustering and immune profiling, while prognostic models were constructed via LASSO/stepwise Cox regression and machine learning optimization.

**Results:**

Three ferroptosis-immune subtypes exhibiting distinct survival outcomes and immune phenotypes were identified. A 40-gene prognostic signature (externally validated) effectively stratified patient survival risk and predicted chemotherapy sensitivity. Single-cell analysis revealed elevated ferroptosis activity within an immunosuppressive microenvironment, specifically implicating POSTN–ITGB5 signaling in fibroblast-immune cell crosstalk. A clinically applicable nomogram integrating risk scores and clinical factors demonstrated robust predictive accuracy (AUC 0.829–0.845). Machine learning refined a 4-gene prognostic signature (CLN6, GMPR, AP1S2, ITGA6), with functional validation confirming the role of CLN6 in proliferation and migration.

**Conclusion:**

This study establishes a prognostic framework and therapeutic roadmap for precision immuno-oncology in melanoma, bridging multi-omics discovery with clinical translation.

## Introduction

Melanoma is the most aggressive form of skin cancer, characterized by high metastatic potential, pronounced heterogeneity, and resistance to conventional therapies. Its global incidence continues to rise, with the prevalence of cutaneous malignant melanoma (CMM) reaching 833,215 cases in 2021—a 161.3% increase since 1990 (1). Although immunotherapies and targeted agents have improved survival outcomes, substantial interpatient variability in treatment response underscores the urgent need for molecular stratification frameworks to guide precision oncology (2, 3).

Ferroptosis, an iron-dependent form of regulated cell death driven by lipid peroxidation, plays dual roles in melanoma progression and therapy resistance (4, 5). Central to this process is the balance between lipid peroxide generation (via PUFA-PL synthesis/extracellular uptake and ACC-mediated biosynthesis) and detoxification by systems like GPX4, which neutralizes phospholipid hydroperoxides (6–13). While ferroptosis susceptibility in dedifferentiated melanoma cells offers therapeutic potential, its context-dependent effects complicate treatment: GPX4/FSP1 preservation in CD8^+^ T cells maintains anti-tumor immunity, whereas ACSL4 deficiency impairs T cell function despite conferring ferroptosis resistance (14–16). Tumor-associated Tregs further evade ferroptosis through reduced lipid peroxidation, sustaining immunosuppression, and MITF downregulation promotes MDSC recruitment, linking melanocyte plasticity to immune evasion (17, 18). These findings highlight the need for strategies that selectively induce ferroptosis in malignant cells while protecting immune effectors to optimize therapeutic outcomes.

Despite advances in characterizing ferroptosis regulators, critical gaps persist in translating these insights into clinical tools. First, the heterogeneity of ferroptosis-related gene expression across melanoma subtypes remains poorly defined, limiting the development of molecularly guided therapies. Second, existing prognostic models often neglect the integration of ferroptosis-immune crosstalk with clinical variables, resulting in suboptimal predictive accuracy. Third, single-cell resolution of ferroptosis activity and its spatial coordination within the tumor immune microenvironment (TME) is lacking, obscuring cell-type-specific vulnerabilities. Additionally, current molecular classifications of melanoma, often based on mutational status (e.g., BRAF, NRAS) or transcriptomic subtypes, insufficiently capture the dynamic crosstalk between regulated cell death pathways and the TME (19, 20). Although single-cell RNA sequencing (scRNA-seq) has advanced our understanding of TME heterogeneity and stromal reprogramming, the spatial and cellular regulation of ferroptosis and its role in intercellular communication remain poorly defined.

This study presents a comprehensive multi-omics characterization of the ferroptosis-immune axis in melanoma. We integrate bulk RNA-seq, single-cell transcriptomics, and machine learning across TCGA and GEO datasets to define ferroptosis-driven molecular subtypes with distinct prognoses and immune landscapes. A core gene set was identified through WGCNA and cross-subtype differential analysis, and a machine learning—based prognostic model was optimized through benchmarking over 100 algorithms. The model was externally validated and linked to immune checkpoint expression, chemotherapy response, and stromal interactions. Through scRNA-seq and CellChat-based ligand-receptor analysis, we further delineate cell type-specific ferroptosis activity and immunoregulatory signaling. Functional validation confirmed the role of CLN6 in proliferation and migration. This integrative approach reveals ferroptosis as a central orchestrator of melanoma heterogeneity, immune escape, and therapeutic vulnerability, offering new avenues for precision stratification and combinatorial targeting strategies.

## Materials and methods

### Data acquisition

GEO datasets: Gene expression data were obtained from the Gene Expression Omnibus (GEO) database (https://www.ncbi.nlm.nih.gov/geo/info/datasets.html). ScRNA-seq data from three melanoma samples were retrieved from GSE215120 for single-cell transcriptome analysis. Bulk RNA-seq data from 69 melanoma samples in GSE53118 and 71 samples in GSE54467 were used for transcriptomic profiling.

TCGA dataset: Processed transcriptomic profiles of 472 melanoma tumor samples were downloaded from The Cancer Genome Atlas (TCGA) via the Genomic Data Commons portal (https://portal.gdc.cancer.gov/). Data types included mRNA expression matrices for downstream analyses.

### Consensus clustering for molecular subtyping

Consensus clustering was conducted to classify melanoma samples based on candidate gene expression levels. Using 50 iterations and subsampling 90% of samples per iteration, we identified the optimal number of clusters via cumulative distribution function (CDF) curves and consensus matrix heatmaps.

### Differential expression analysis

Differential gene expression between melanoma and control samples was assessed using the limma package in R. Genes with adjusted *p-values < 0.05* and |log2 fold change| > 0.585 were considered significantly differentially expressed. Volcano plots were generated for visualization.

### Functional enrichment analysis of molecular subtypes

Functional pathway enrichment across identified subtypes was evaluated using single-sample gene set enrichment analysis (ssGSEA) implemented via the *GSVA* R package. GO and KEGG gene sets were sourced from MSigDB (v7.5.1): c2.cp.kegg.v7.5.1.symbols and c5.go.v7.5.1.symbols.

### Immune infiltration analysis

Immune cell composition was inferred using the CIBERSORT algorithm (21), a support vector regression-based method for deconvolution of bulk expression profiles. Using 547 signature genes, CIBERSORT quantified 22 immune cell types, including T cells, B cells, plasma cells, and myeloid subtypes. Pearson correlation analysis assessed associations between gene expression and immune cell proportions.

### Weighted gene co-expression network analysis

Gene co-expression networks were constructed using the WGCNA R package (22). We selected the top 10,000 most variable genes by variance for analysis. A soft-thresholding power of 8 was applied to approximate scale-free topology. The adjacency matrix was transformed into a topological overlap matrix (TOM), followed by hierarchical clustering to identify gene modules. Module eigengenes were correlated with risk scores to determine biologically relevant modules.

### Machine learning–based prognostic model construction

A multi-algorithm screening process was used to construct and validate prognostic models based on candidate genes. Model development employed the Mime1 R package and the ML.Dev.Prog.Sig function, incorporating over 100 machine learning algorithms. Model performance was evaluated via concordance index (C-index) distribution and Kaplan–Meier survival analyses. External validation was performed on independent datasets.

### Drug sensitivity analysis

Chemotherapy response was forecast using the oncoPredict R package and training datasets sourced from the Genomics of Drug Sensitivity in Cancer (GDSC) database (https://www.cancerrxgene.org/). Half-maximal inhibitory concentrations (IC50s) were determined using a regression model that incorporated 10-fold cross-validation. Batch effects were corrected using the “combat” method, and low-variance and duplicated genes were filtered before modeling.

### Gene set enrichment analysis

GSEA was performed to identify differentially enriched pathways between high- and low-risk groups. Gene sets from MSigDB v7.0 were used as the background. Pathways with an adjusted *p-value < 0.05* were deemed significantly enriched and ranked by normalized enrichment scores (NES).

### Gene set variation analysis

GSVA, an unsupervised, non-parametric enrichment method, was used to assess variation in pathway activity across samples. Gene sets from MSigDB were used as references. GSVA scores were calculated for each sample and pathway to evaluate functional state changes.

### Nomogram construction

A final cohort of 337 melanoma samples from TCGA with complete clinical annotations was analyzed. A multivariable Cox regression model was used to construct a nomogram integrating clinical features and gene expression. Each variable was assigned a weighted score based on its regression coefficient, and the total score was mapped to predicted survival probabilities.

### Single-cell quality control

Quality control for scRNA-seq data was performed using the *Seurat* R package. Filtering criteria included thresholds for total UMI counts, gene counts per cell, and mitochondrial/ribosomal gene content. Cells exceeding three median absolute deviations (MADs) from the median were excluded. DoubletFinder (v2.0.4) was used to remove doublets.

### Single-cell data normalization and processing

Data normalization was performed using *NormalizeData* and identifying highly variable genes via *FindVariableFeatures*. The data were scaled with *ScaleData* to regress out mitochondrial/ribosomal gene effects and cell cycle variation. Dimensionality reduction was performed using PCA (*RunPCA*) and batch correction using *Harmony*. Clustering was conducted via FindClusters, and cell annotation was performed using CellMarker, literature curation, and the *SingleR* tool.

### Ligand–receptor interaction analysis

Intercellular communication was inferred using CellChat (23), a computational framework for analyzing ligand-receptor networks from scRNA-seq data. Standardized expression matrices and cell subtype annotations were input. Interaction strength and frequency were quantified, and communication networks were mapped to assess signaling patterns associated with risk phenotypes.

### Random survival forest

The RSF, a machine learning algorithm designed for survival data analysis, was applied to screen feature genes and rank their prognostic importance. Using the randomForestSRC package in R, we implemented the RSF algorithm with nrep = 1000 (indicating 1000 iterations in Monte Carlo simulations for stability assessment). Genes were ranked based on their variable importance scores, and those with a relative importance > 0.2 were selected as final candidate feature genes.

### Cell culture, RNA extraction, and qRT-PCR

The human melanoma cell lines A375 (Stem Cell Bank, Chinese Academy of Sciences) and SKMEK28 were cultured in DMEM (GIBCO, USA) containing 10% fetal bovine serum (FBS) (GIBCO, USA), 1% penicillin-streptomycin (100 U/mL penicillin and 100 μg/mL streptomycin), and 5% CO2 in an incubator at 37 °C, while normal human epithelial keratinocytes (NHEK) were maintained in DMEM/F12 (GIBCO, USA) supplemented with 10% FBS and 10 ng/mL epidermal growth factor (EGF) and 1% penicillin-streptomycin. The manufacturer’s protocol extracted Total RNA using TRIzol (Invitrogen). RNA concentrations were measured with a Nanodrop 2000C (Thermo Fisher Scientific), and cDNA was synthesized using a ThermoFisher reverse transcription kit. qRT-PCR was performed in a 20 µL reaction volume containing SYBR Green Master Mix (CW0957H, Kangwei) and gene-specific primers. The reaction conditions included 95°C for 10 min followed by 45 cycles at 95 °C for 10 s and 60 °C for 30 s. Primer sequences for CLN6, GMPR, AP1S2, ITGA6, and the β-actin reference gene are listed in detail below. The gene primer sequences were: Forward 5’- TGCCATGCTGGTATTCCCTC-3’, reverse 5’ - TGATGACGTTGTAGGCCATGT-3’ for human CLN6; Forward 5’- CTCAAGCTCGACTTCAAGGATG-3’, reverse 5’- GGGAATCCCTGAGTAGGTCTG-3’ for human GMPR; Forward 5’- TTCAGACCGTTTTAGCACGGA-3’, reverse 5’- TGTCCTGATCCTCAATAGCACA-3’ for human AP1S2; Forward 5’- GGCGGTGTTATGTCCTGAGTC -3’, reverse 5’- AATCGCCCATCACAAAAGCTC-3’ for human ITGA6; Forward 5’-CACCAACTGGGACGACAT-3’, reverse 5’- ACAGCCTGGATAGCAACG-3’ for human β-actin. Relative expression levels were normalized to β-actin. The siRNA was purchased from GenePharma (Shanghai, China). siRNA sequences were as follows: siCLN6-1: sense: 5’-GACCUCUGGUUCUACUUCATT-3’, antisense: 5’-UGAAGUAGAACCAGAGGUCTT-3’; siCLN6-2: sense: 5’-GAACCCCAUCAUCAAGAAUTT-3’, antisense: 5’-AUUCUUGAUGAUGGGGUUCTT-3’. Cells were incubated in 6-well plates, and transfection was started when cell density reached 60%. Transfection was performed using Lipofectamine RNAiMAX Transfection Reagent (Thermo Fisher Scientific) in all cells maintained in Opti-MEM. The culture medium was replaced with fresh medium supplemented with 10% FBS after 12 h post-transfection. Transfection efficiency was detected using qRT-PCR.

### MTS assay

A cell proliferation assay was performed in 96-well plates, 1000 cells were added to each well after counting. 24, 48, 72, 96 h later, 10 μL MTS solution (3-(4, 5-dimethylthiazol-2-yl)-5-(3-carboxymethoxyphenyl)-2-(4-sulfophenyl)-2H tetrazolium) (Promega, Madison, WI, USA) was added to each well, incubated for 3 h, and the absorbance at 495 nm (OD) was detected by an enzyme labeling instrument (Thermo Fisher Scientific) according with the manufacturer’s guidelines.

### Wound healing assay

Wound healing assay was used to assess the migration ability of the cells. Transfected cells (10× 10^4^/well) were inoculated in 6-well plates with a monolayer of cells evenly distributed on the bottom of the plate. The cell layer was scraped with a 200 μl pipette tip, washed with PBS, and FBS-free medium was added to each well. Images were then taken under an inverted microscope at 0 and 24h.

### Statistical analysis

All experimental analysis was represented as the mean ± standard deviation (SD) from three times of replicative experiments. Microsoft Excel and GraphPad Prism 5 were used to draw the charts. All statistical analyses were conducted in R version 4.3.0. A *p-value < 0.05* was considered statistically significant unless stated otherwise.

## Results

### Molecular subtyping based on ferroptosis-associated gene expression

**Figure 1:**
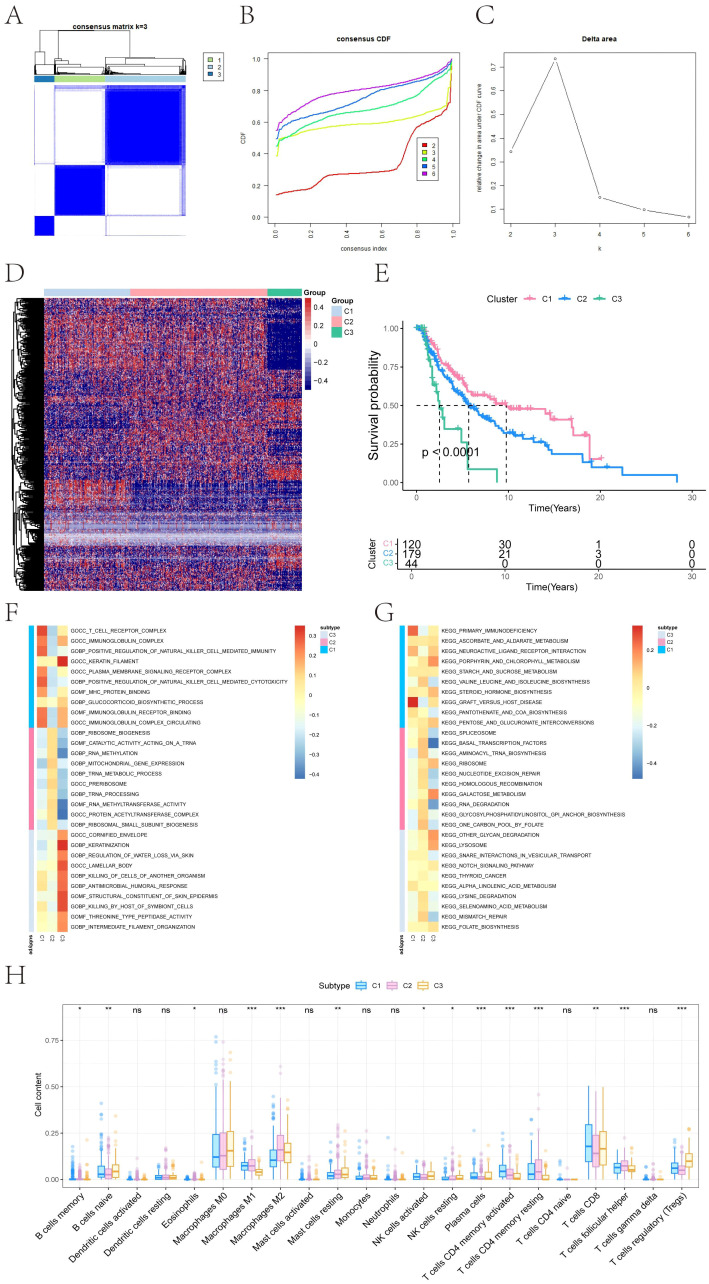
Consensus clustering–based molecular subtyping and survival analysis of melanoma. **(A)** Consensus matrix heatmap showing robust subtype separation (C1, C2, C3) based on 484 ferroptosis-related genes from FerrDb. **(B)** Cumulative distribution function (CDF) curves for consensus clustering (K = 2–6). **(C)** Delta area plot indicating optimal cluster number (K = 3). **(D)** Heatmap displaying the expression of ferroptosis-related genes across clusters C1, C2, and C3. **(E)** Kaplan–Meier curves showing significant differences in overall survival (OS) among clusters. Statistical significance was determined by the Log-rank test (*P < 0.0001*). C1 exhibits the most favorable prognosis, while C3 correlates with poor clinical outcomes. **(F, G)** Gene Ontology (GO) and Kyoto Encyclopedia of Genes and Genomes (KEGG) pathway enrichment analysis for each subtype, highlighting distinct immune, mitochondrial, and lysosomal signatures. Red indicates high expression, and blue indicates low expression. **(H)** Box plots summarizing immune cell infiltration differences across clusters based on CIBERSORT deconvolution. Significance was assessed using ANOVA, which is specifically designed for multi-group comparisons (*P < 0.05, **P < 0.01, ***P < 0.001).

Ferroptosis is closely associated with melanoma and can be leveraged as a potential therapeutic strategy to overcome drug resistance, enhance the efficacy of chemotherapy and radiotherapy, and modulate the tumor immune microenvironment. Thus, we obtained 484 ferroptosis-related genes from the FerrDb database (http://www.zhounan.org/ferrdb) and performed consensus clustering to stratify melanoma patients based on their expression profiles (**Figures 1A–C**). The optimal number of clusters was determined as K = 3, resulting in three molecular subtypes: C1 (N=158), C2 (N=251), and C3 (N=63). A heatmap illustrated distinct gene expression patterns across these subtypes (**Figure 1D**). Survival analysis revealed significant differences in overall survival (OS) among the clusters (log-rank *P < 0.0001*), with cluster C1 showing the best prognosis and cluster C3 the worst (**Figure 1E**).

### Pathway enrichment profiles define subtype-specific biological features

To investigate the functional characteristics of each subtype, GSEA was performed. Subtype C1 exhibited enrichment in immune-related pathways, including T cell receptor complex, immunoglobulin complex gene sets (GO), and KEGG terms such as primary immunodeficiency and graft-versus-host disease. C2 showed upregulation of mitochondrial and translational programs, including mitochondrial gene expression and aminoacyl-tRNA biosynthesis. C3 was enriched for epithelial differentiation and lysosomal activity, including keratinization and glycan degradation (**Figures 1F, G**). These results confirm that each ferroptosis-immune subtype exhibits distinct molecular and functional signatures.

### Immune landscape differences across ferroptosis subtypes

Immune infiltration analysis revealed subtype-specific variations in the tumor immune microenvironment. Significant differences were observed in the relative abundance of memory B cells, naive B cells, eosinophils, macrophage subtypes (M1/M2), resting mast cells, NK cells (activated/resting), plasma cells, CD4+ and CD8+ T cell subsets, follicular helper T cells, and regulatory T cells (Tregs) (**Figure 1H**). These findings highlight the immunological heterogeneity among ferroptosis-related molecular subtypes.

### Differential gene expression across subtypes

**Figure 2:**
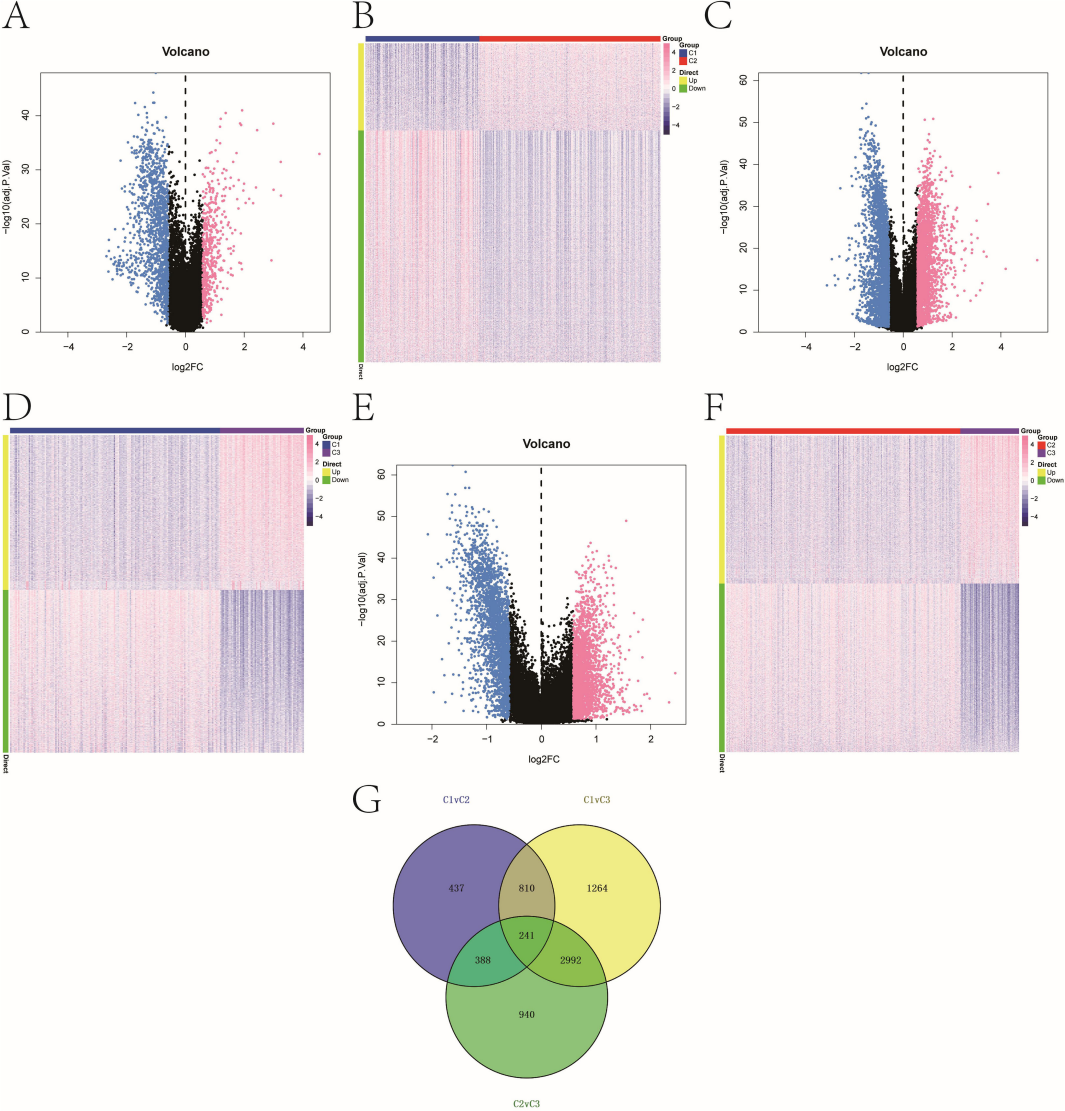
Melanoma subtype-specific gene expression. **(A–F)** Volcano plots and heatmaps showing DEGs between: **(A, B)** C1 vs. C2; **(C, D)** C1 vs. C3; **(E, F)** C2 vs. C3. Thresholds: |log2FC| > 0.585, adjusted *P < 0.05*. The color scale of heatmaps representing high expression in red and low expression in blue. **(G)** Venn diagram illustrating 241 overlapping DEGs common to all three pairwise comparisons.

Pairwise differential expression analysis using *limma* on TCGA data identified 1,876 DEGs between C1 and C2, 5,307 DEGs between C1 and C3, and 4,561 DEGs between C2 and C3 (|logFC| > 0.585, adjusted *P* < 0.05). Volcano plots and heat maps illustrated the distribution of DEGs (**Figures 2A–F**). A Venn diagram identified 241 overlapping DEGs shared across all three comparisons, forming a core candidate gene set for downstream modeling (**Figure 2G**).

### Integrating DEGs with WGCNA modules to identify hub genes

**Figure 3:**
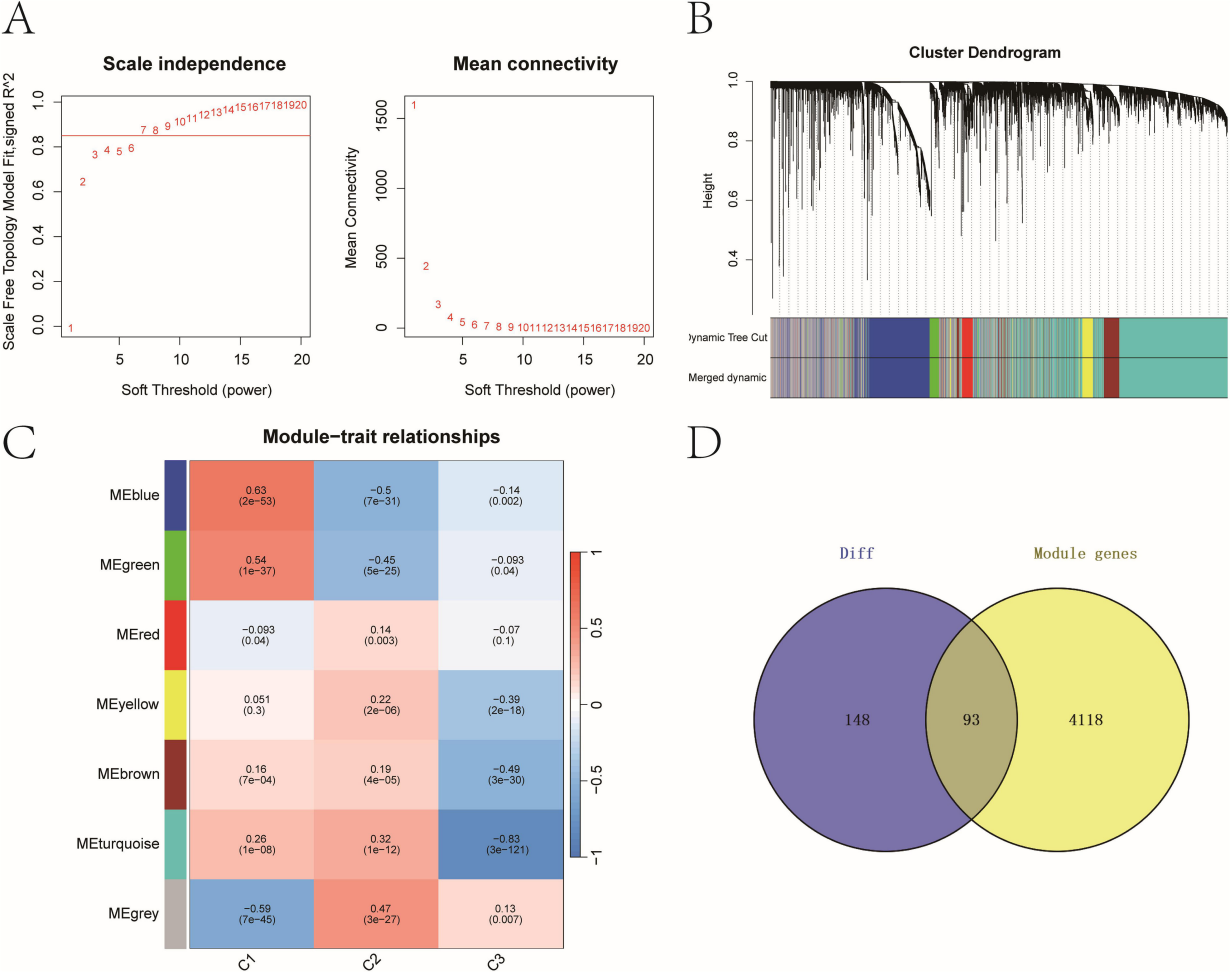
Key prognostic genes with strong differential expression and co-expression patterns. **(A)** Scale-free topology model fit and mean connectivity plot used to select soft-thresholding power (β = 7). **(B)** Dendrogram of gene co-expression modules with merged color-coded branches. **(C)** Module-trait correlations between WGCNA modules and molecular subtypes. Heatmap of module–trait correlations showing strongest association between the turquoise module and cluster C3 (cor = –0.83, *P = 3e−121*). **(D)** Venn diagram identifying 93 overlapping genes between the 241 DEGs and 4,207 genes in the turquoise module.

WGCNA was used to construct gene co-expression networks. Seven modules were identified with a soft threshold (β = 7) (**Figures 3A–C**). The turquoise module (n = 4,207) exhibited the strongest negative correlation with cluster C3 (cor = -0.83, *P* = 3e-121). Intersecting this module with the 241 core DEGs yielded 93 overlapping genes (**Figure 3D**), which were prioritized as high-confidence prognostic candidates (see **Supplementary Table 1**).

### Prognostic model optimization using machine learning

**Figure 4:**
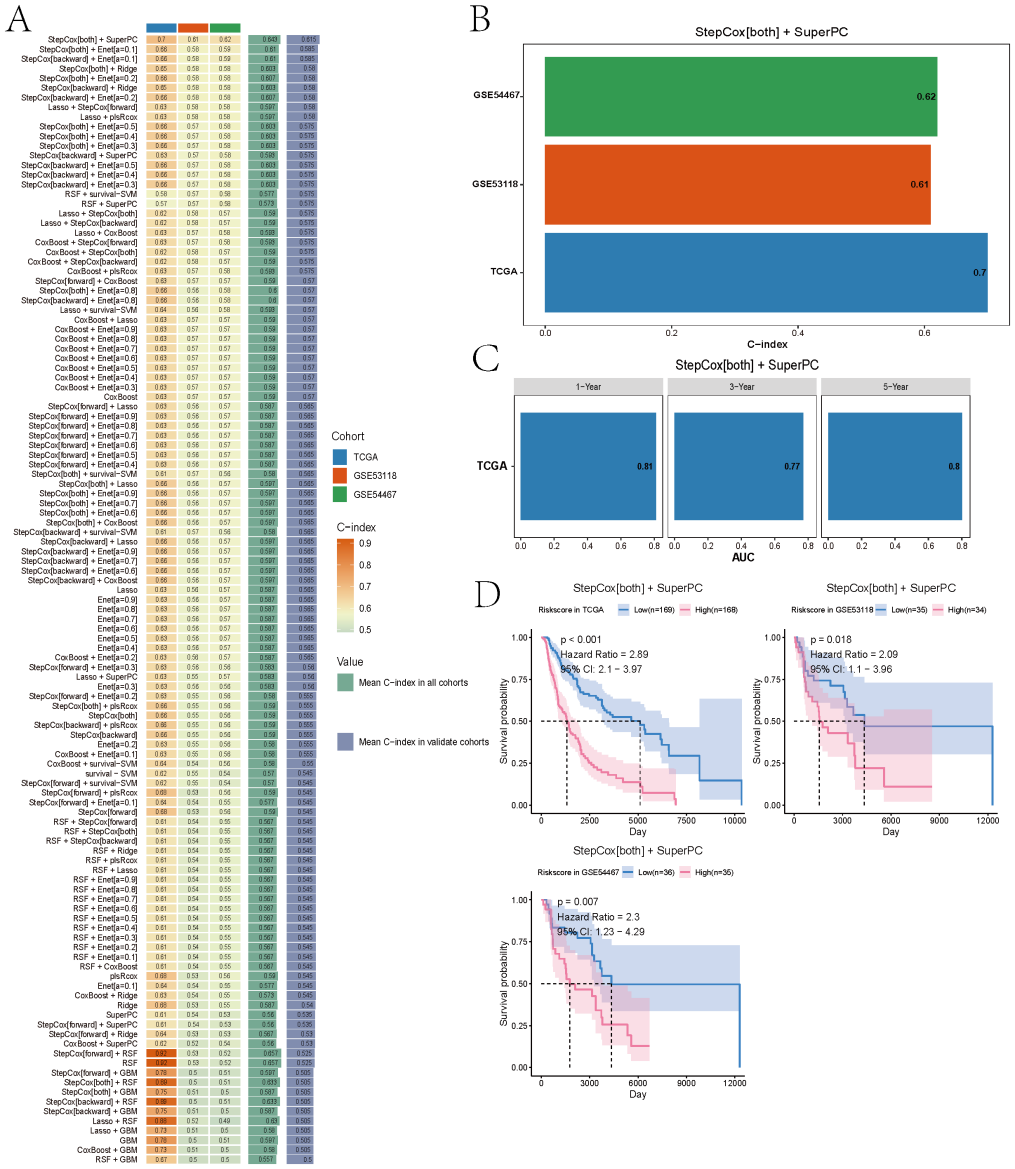
Selecting a best melanoma prognostic model via machine learning. **(A)** C-index distribution across 100+ machine learning algorithms. **(B)** Performance evaluation of 100+ machine learning algorithms identifying StepCox[both] + SuperPC as optimal based on C-index across datasets. **(C)** Time-dependent ROC curves evaluating predictive accuracy at 1, 3, and 5 years in TCGA set (AUCs: 0.81, 0.77, 0.8). The AUC values range between 0.5 and 1, where 0.5 indicates no discriminative ability and 1 represents perfect discriminative ability. **(D)** Kaplan–Meier survival curves showing significant OS differences between high- and low-risk groups in training and validation cohorts.

We constructed prognostic models using the 93 candidate genes via benchmarking over 100 machine-learning algorithms. The final set of 40 genes (see **Supplementary Table 1**) incorporated into the model showed significant functional enrichment in pigment metabolism, apoptotic signaling, and specific cellular structures (**Supplementary Image 2**). The StepCox^[both]^ + SuperPC model demonstrated the best performance, with C-index values of 0.7, 0.61, and 0.62 in TCGA and two GEO validation cohorts, respectively (**Figures 4A, B**). ROC analysis showed AUCs of 0.81, 0.77, and 0.8 for 1-, 3-, and 5-year survival prediction, respectively (**Figure 4C**). Stratification based on the model’s risk score revealed significant OS differences in both training and validation cohorts (log-rank *P* < 0.05, **Figure 4D**).

### Immunotherapy response prediction and TME associations

**Figure 5:**
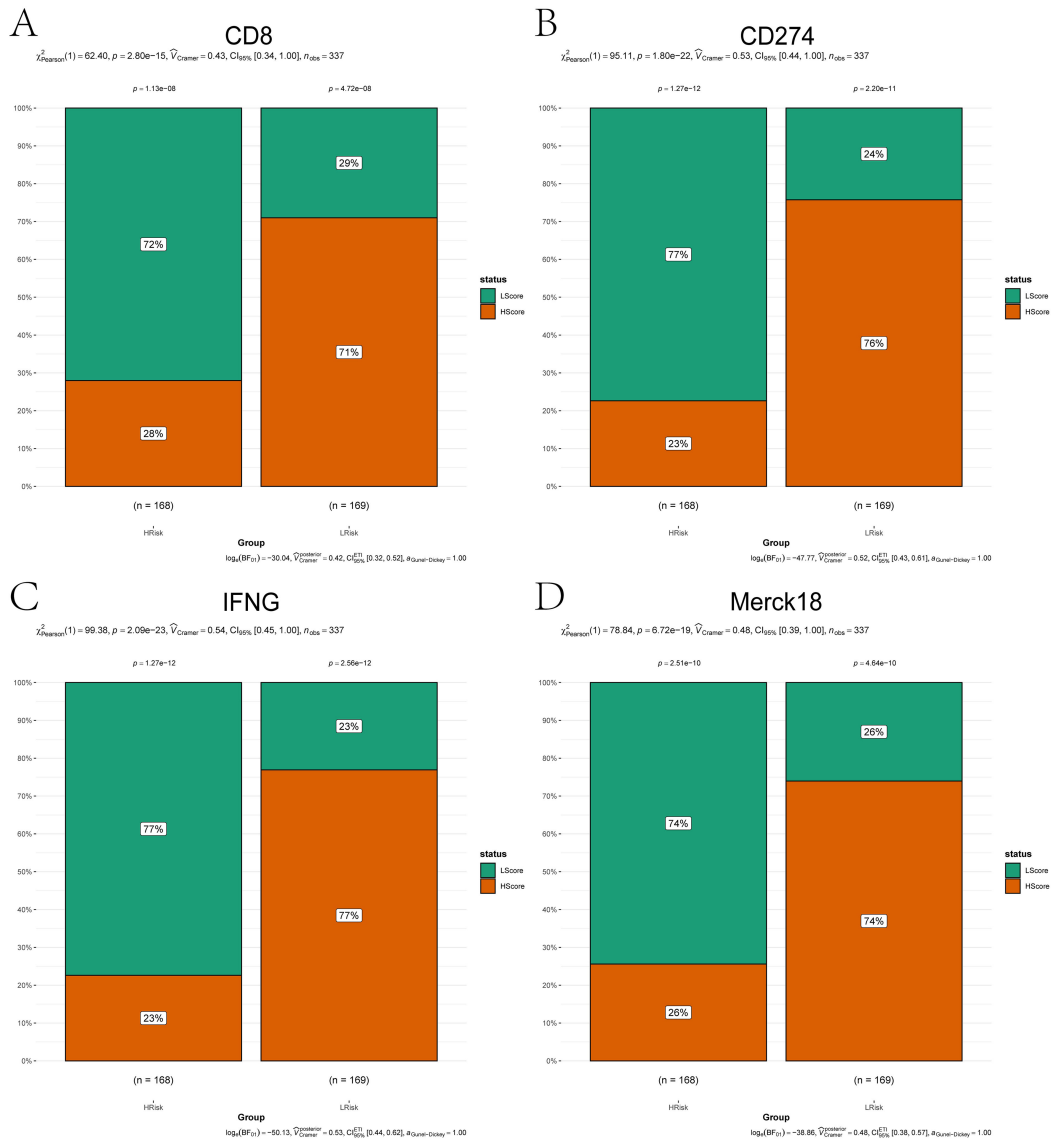
Immunotherapy biomarker expression across risk subgroups. **(A–D)** Bar plots comparing expression levels of immunotherapy-related markers between high-risk (orange) and low-risk (green) groups: **(A)** CD8; **(B)** CD274; **(C)** IFNG; **(D)** Merck18. Categorical associations (e.g., immune responder vs. non-responder status) were statistically validated using the Chi-squared test (χ², *P* < 0.05).

**Figure 6:**
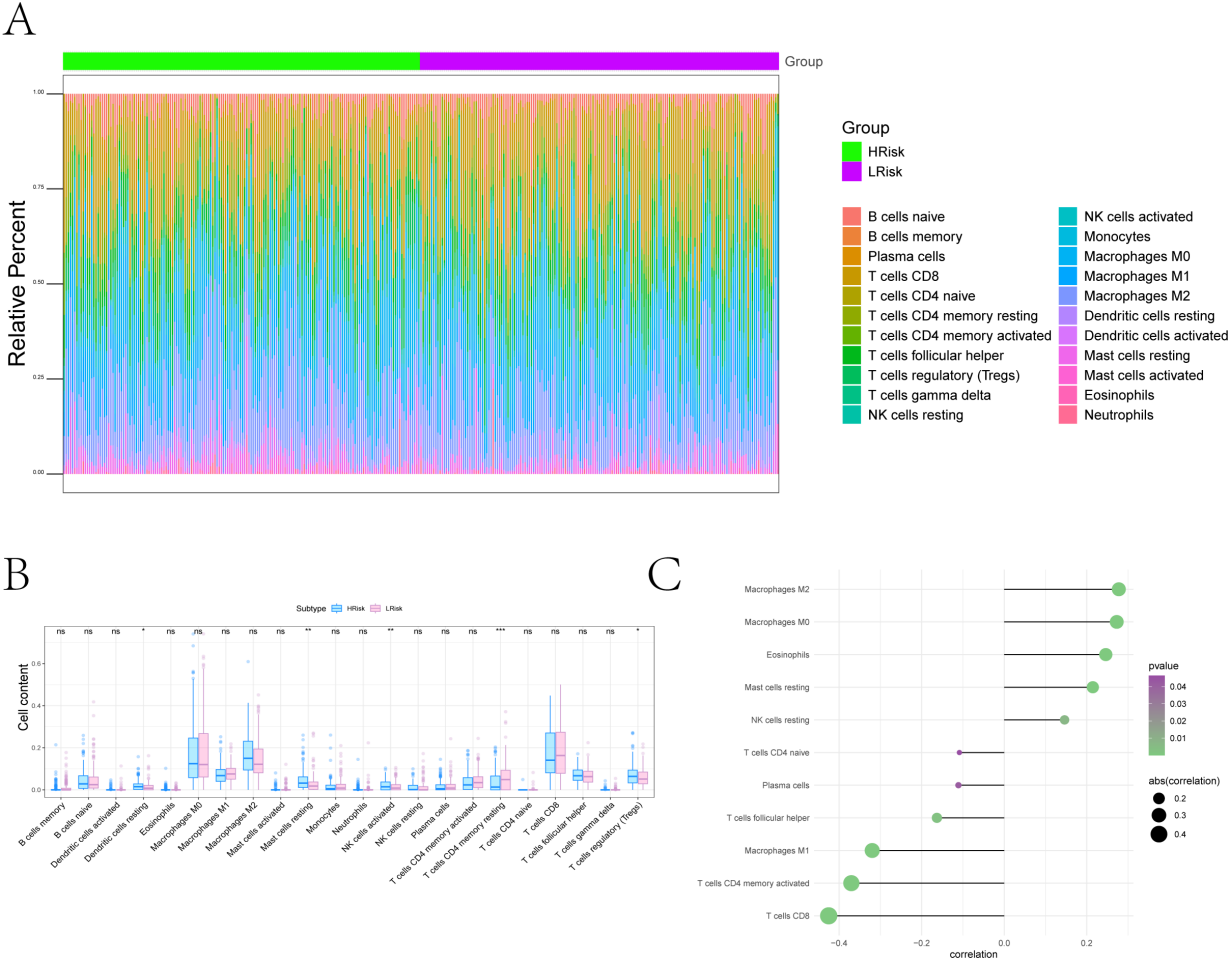
Tumor microenvironment remodeling associated with the ferroptosis-based risk score. **(A)** Overall immune landscape comparisons between risk groups using CIBERSORT. **(B)** Boxplots of differentially abundant immune cell types (FDR < 0.05) (*P < 0.05, **P < 0.01, ***P < 0.001). **(C)** Lollipop plot illustrating associations between risk score and immune cell populations: Positive correlations with Resting dendritic cells, Resting Mast cells, Eosinophils, Macrophages M0, and Macrophages M2; negative correlations with Naive CD4^+^ T cells, Plasma cells, T cells follicular helper, Macrophages M1, Activated CD4^+^ memory T cells, and CD8^+^ T cells.

To analyze the association between risk stratification and immunotherapy response/immune evasion mechanisms, we leveraged the TIDE algorithm to infer functional states of immune cells (e.g., T-cell dysfunction, macrophage immunosuppression) from transcriptomic profiles of high- and low-risk groups. Expression levels of immunotherapy-related biomarkers (Merck18, CD8, CD274, IFNG) significantly differ between high- and low-risk groups (**Figures 5A–D**). Among the four groups, the high-risk group showed a higher proportion of low-risk score (LScore) than high-risk score (HScore), whereas the low-risk group exhibited the opposite pattern (HScore > LScore). This indicates that immune response status may be associated with risk stratification, with high-risk groups potentially linked to diminished immune activation and low-risk groups correlating with enhanced immune responsiveness. Immune infiltration analysis showed notable differences in resting dendritic cells, mast cells, NK cells, and CD4+ memory T cells between risk groups (**Figures 6A, B**). Correlation analyses revealed positive associations between risk score and activated NK cells, resting dendritic cells, mast cells, and Tregs, and negative associations with resting CD4+ memory T cells (**Figure 6C**). While weak correlations (|r| ≈ 0.2) require cautious interpretation, they remain biologically plausible in heterogeneous tumors. These results support the model’s relevance to immune landscape remodeling.

### Multi-dimensional biomarker analysis: immune regulation and drug sensitivity

**Figure 7:**
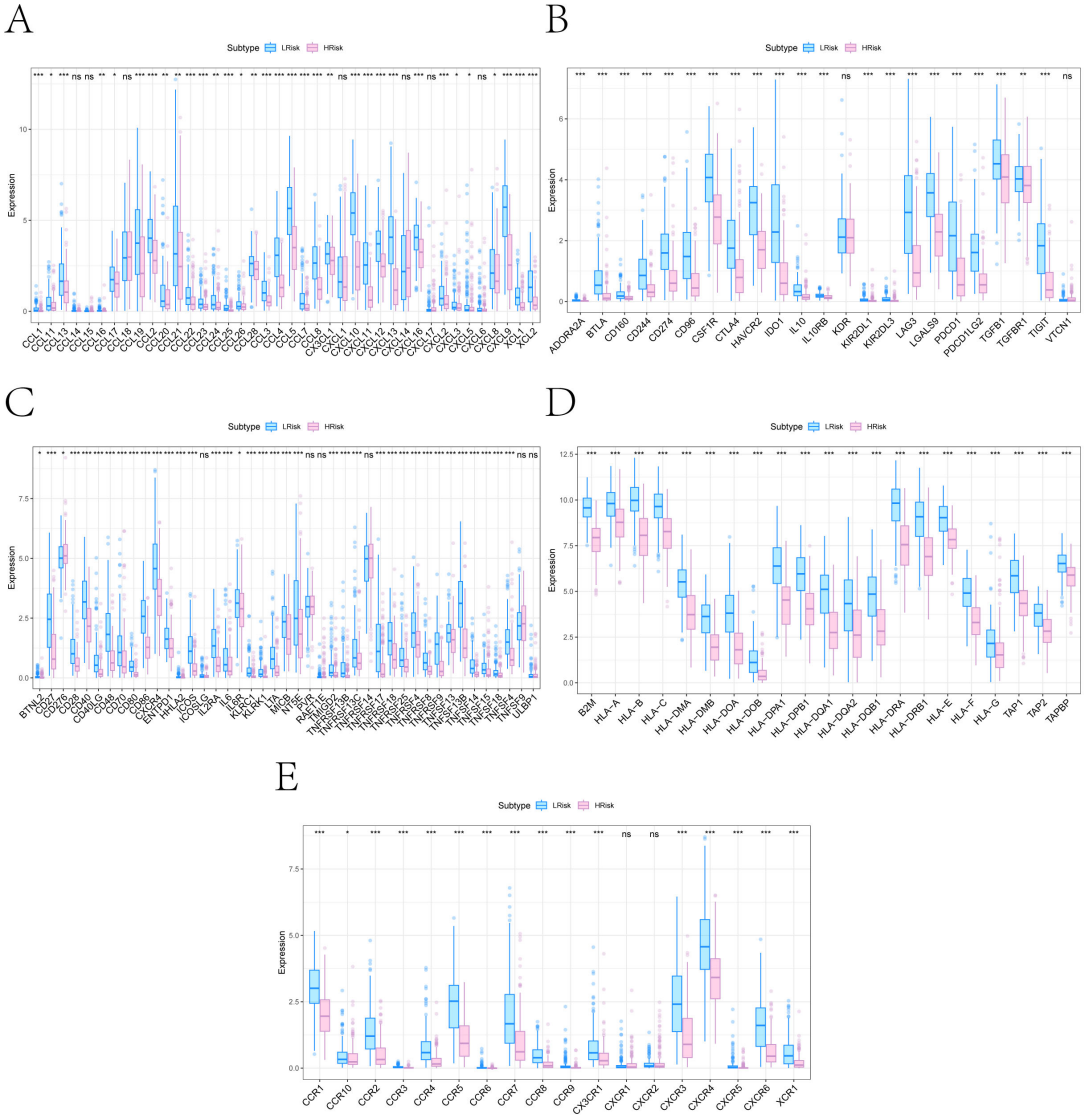
Immune regulatory gene differences between high- and low-risk subgroups. **(A–E)** Box plots showing expression differences in five immune regulatory gene categories retrieved from the TISIDB database: **(A)** Chemokines; **(B)** Immunoinhibitors; **(C)** Immunostimulators; **(D)** MHC molecules; **(E)** Chemokine receptors (*P < 0.05, **P < 0.01, ***P < 0.001).

**Figure 8:**
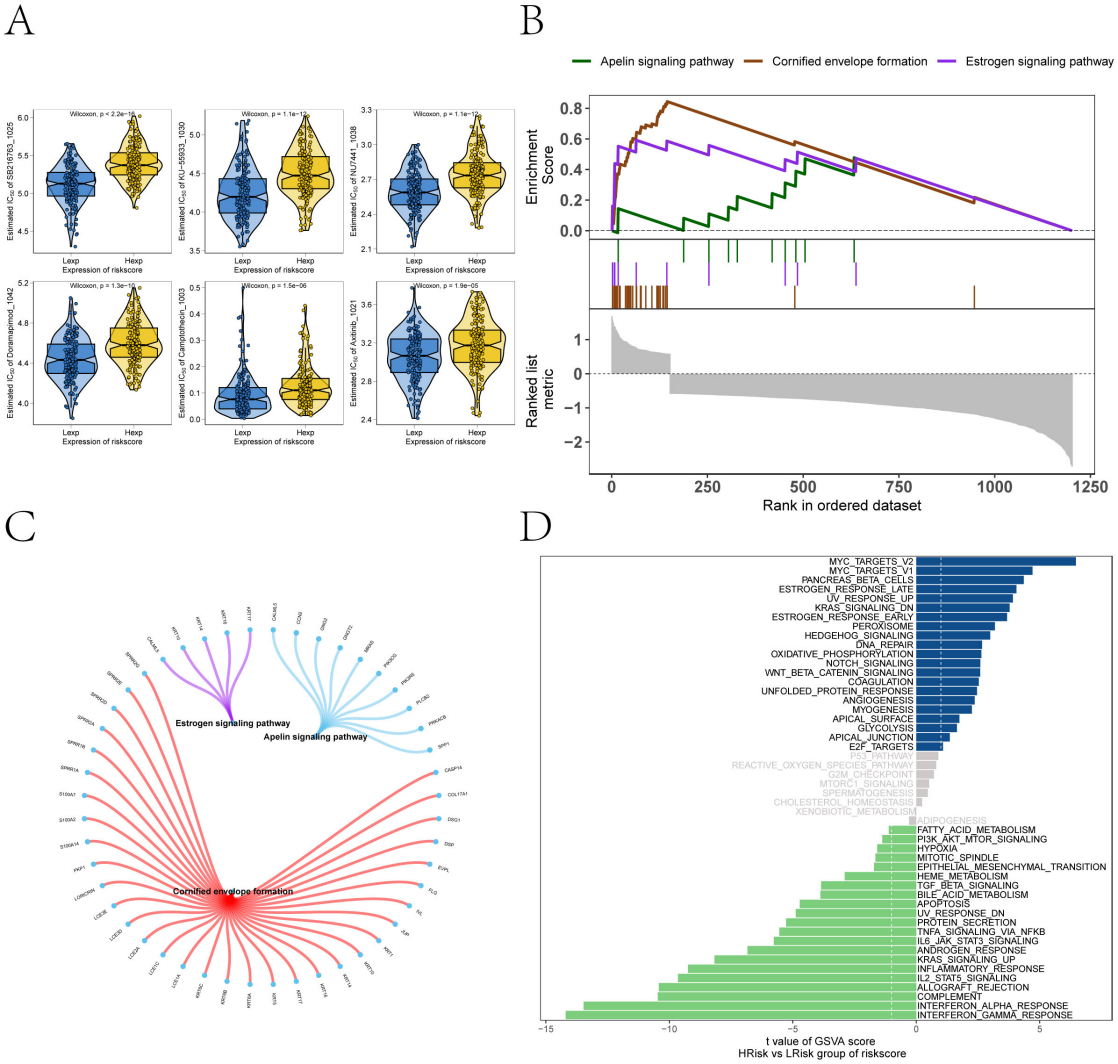
Drug sensitivity and signaling pathway activation contrasting the high- and low-risk subgroups. **(A)** Predicted sensitivity to chemotherapeutic agents via oncoPredict, showing risk score associations with SB216763, KU-55933, NU7441, Doramapimod, Camptothecin, and Axitinib. Low-risk patients exhibit heightened sensitivity to SB216763, KU-55933, NU7441, Doramapimod, Camptothecin, and Axitinib (Wilcoxon *P≤ 0.05*). **(B)** GSEA pathway enrichment analysis of high- versus low-risk groups. **(C)** The molecular interaction network cross pathways. **(D)** GSVA highlighting distinct enrichment of pathways (MYC_TARGETS_V2, MYC_TARGETS_V1, OXIDATIVE_PHOSPHORYLATION) in high- versus low-risk groups.

We conducted subsequent analyses to explore the associations between the risk score and immunomodulators, as well as chemotherapy sensitivity. Differential analysis of immunomodulators from the TISIDB database revealed subtype-specific expression patterns among immunosuppressive genes, immunostimulants, chemokines, and receptors (**Figures 7A–E**). Drug sensitivity prediction using *oncoPredict* showed significant associations between the risk score and sensitivity to several agents (**Figure 8A**). The low-risk score group exhibited lower IC50 values for SB216763, KU-55933, NU7441, Doramapimod, Camptothecin, and Axitinib (*P ≤ 0.05*), indicating heightened sensitivity to these agents. These findings provide clearer therapeutic selection criteria for melanoma based on risk stratification.

### Pathway enrichment underlying the risk model

GSEA revealed significant pathway associations with the risk score, including positive enrichment of Apelin signaling pathway, Cornified envelope formation, and Estrogen signaling pathway (**Figures 8B, C**). GSVA analysis supported these findings, revealing enrichment of MYC_TARGETS_V2, MYC_TARGETS_V1, OXIDATIVE_PHOSPHORYLATION, and WNT_BETA_CATENIN_SIGNALING in high-risk groups, and negative enrichment of FATTY_ACID_METABOLISM and PI3K_AKT_MTOR_SIGNALING (**Figure 8D**). Samples with high-risk scores showed enrichment in pathways corresponding to the blue region, whereas those with low-risk scores were enriched in pathways associated with the green region. These results suggest that the risk score reflects coordinated reprogramming of metabolic and inflammatory signaling networks.

### Prognostic nomogram development

**Figure 9:**
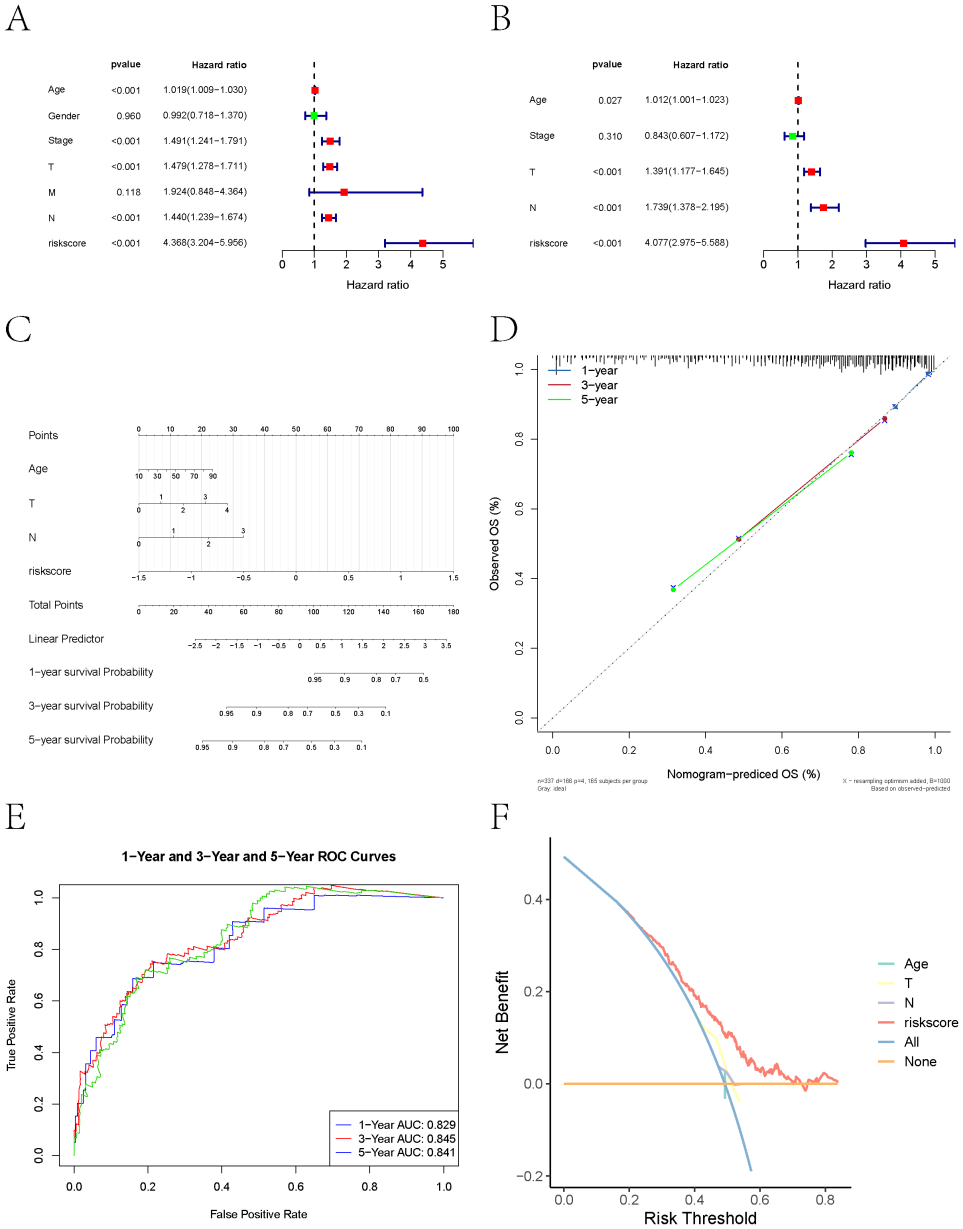
Nomogram-based prognostic modeling. **(A)** Forest plot of univariate Cox regression analysis of clinical data and key gene expression levels in melanoma patients. **(B)** Key clinical indicators (Age, T stage, N stage, and risk score) identified through multivariate Cox regression analysis (*P<0.05*). **(C)** Nomogram combining clinical variables and risk score developed using multivariable Cox regression. Points assigned to each variable are summed to calculate total risk, mapped to survival probability on the bottom axis. **(D)** Calibration plots showing predicted vs. observed OS at 1, 3, and 5 years. Diagonal dashed line represents perfect concordance. **(E)** ROC analysis demonstrating predictive performance. AUC values of 0.829 (1-year), 0.845 (3-year), and 0.841 (5-year) confirm robust discriminative capacity. Shaded regions denote 95% confidence intervals. **(F)** Decision curve analysis (DCA) indicating net clinical benefit over single-variable models.

Based on clinical data and key gene expression levels, we established univariate Cox regression models and generated forest plots (**Figure 9A**). Subsequently, factors with a *p-value* less than 0.05 were extracted for multivariate analysis. The results revealed that Age, T stage, N stage, and risk score had *p-values* less than 0.05 (**Figure 9B**). We therefore identified these as key clinical indicators and proceeded to construct a nomogram model. Using the risk score, we presented the results of the regression analysis in the form of a nomogram. The regression analysis indicated that the values of different clinical indicators for melanoma and the distribution of the risk score expression contribute variably to the overall scoring process.

Furthermore, this study performed predictive analyses for overall survival (OS) at 1-year, 3-year, and 5-year time points. The results demonstrated a close agreement between the predicted OS and the observed OS, indicating that the nomogram model possesses good predictive performance (**Figures 9C, D**). ROC curves and DCA curves were also generated (**Figures 9E, F**).

### Single-cell quality control and integration

After filtering low-quality or doublet cells (UMIs < 200 or >3 MAD), 24,111 high-quality cells remained (**Supplementary Images 1A, B**). Quality metrics were visualized using violin and scatter plots. HVG selection (n = 2,000) was followed by normalization, scaling, PCA, and Harmony-based batch correction to produce an integrated dataset for clustering (**Supplementary Images 1C–F**). To tackle potential batch effects, we first applied the Harmony algorithm and then performed nonlinear dimensionality reduction using RunUMAP (Uniform Manifold Approximation and Projection).

### Cell type annotation and TME composition

**Figure 10:**
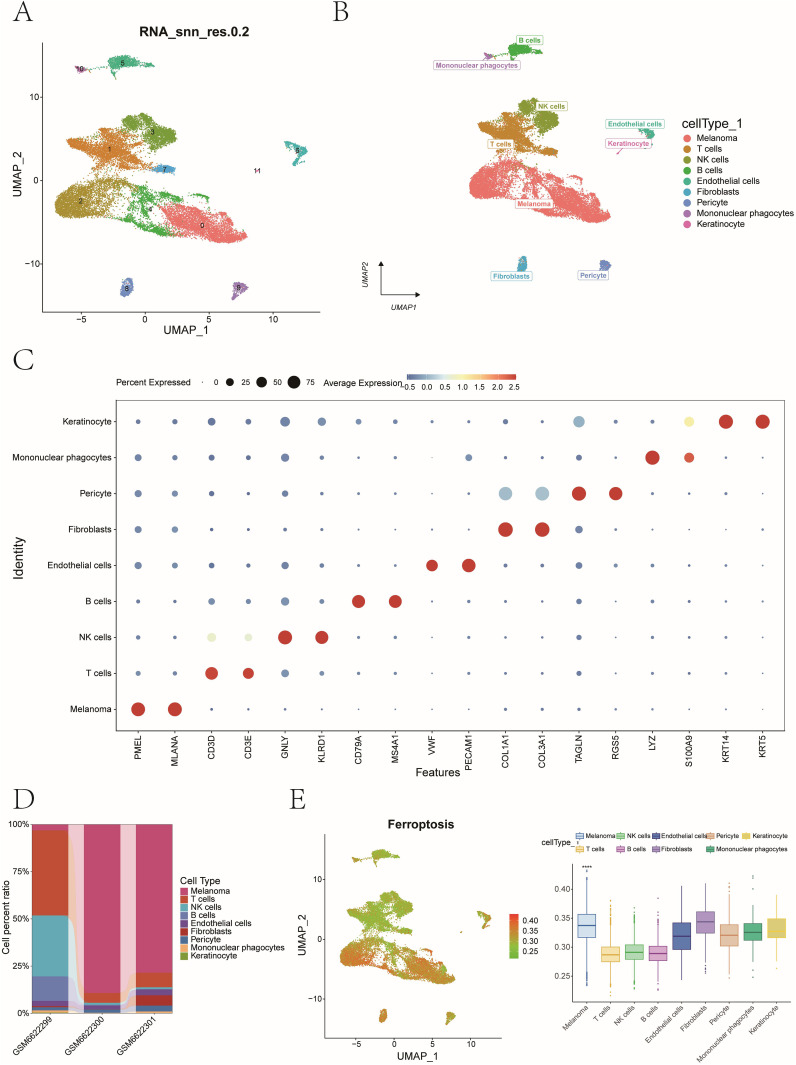
Single-cell clustering, cell annotation, and ferroptosis activity quantification. **(A)** UMAP dimensionality reduction and Leiden algorithm - based cell clustering identified 12 distinct cell clusters. **(B)** Cell type annotation using canonical marker genes. Marker genes defining by CellMarker and literature-curated signatures. **(C)** A dot plot showing expression frequency and levels for lineage-specific markers. **(D)** Stacked bar plots showing cell type proportions across patient samples. **(E)** AUCell quantification of ferroptosis activity at single-cell resolution (****P<0.0001).

After UMAP dimensionality reduction, cell clustering using the Leiden algorithm resolved 12 distinct clusters (**Figure 10A**). These clusters were annotated into nine major cell populations—Melanoma, T cells, NK cells, B cells, Endothelial cells, Fibroblasts, Pericyte, Mononuclear phagocytes, and Keratinocytes—using marker genes defined by CellMarker and literature-curated signatures (**Figure 10B**). Marker gene expression across nine cell types was visualized via bubble plots (**Figure 10C**), while bar plots depicted sample-specific cellular proportions for these nine cell types (**Figure 10D**).

### Ferroptosis activity and ligand–receptor interactions

**Figure 11:**
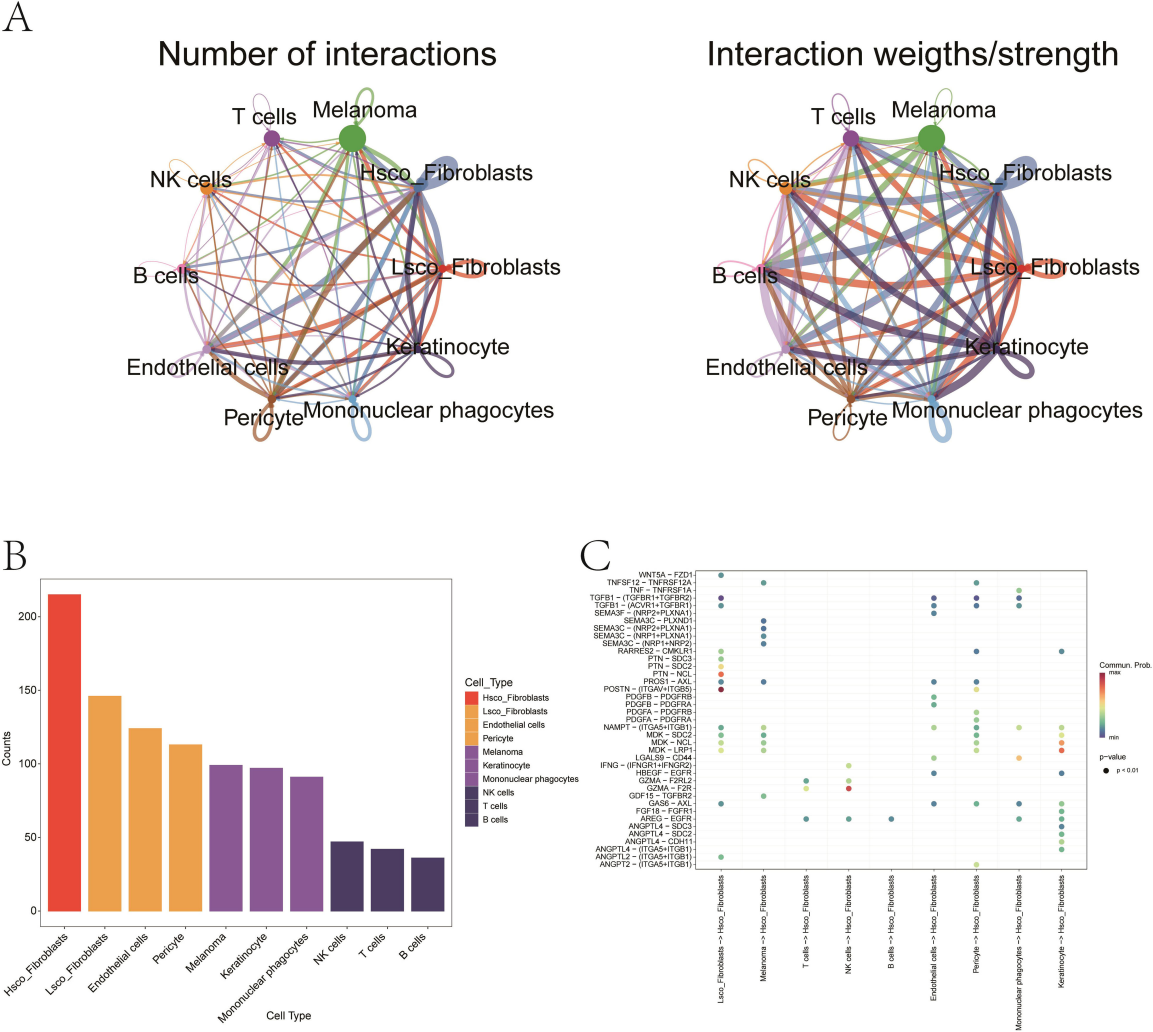
Ligand–receptor interactions mediating fibroblast–melanoma crosstalk in ferroptosis context. **(A)** The network illustrating quantitative connectivity patterns among distinct cellular subpopulations, with line width representing interaction strength. Node size corresponds to cell population abundance, and edge color denotes directionality (from sender to receiver cells). **(B)** Bar plot showing the distribution of distinct cellular subpopulations. **(C)** Bubble chart representing the probability of signaling via specific ligand–receptor pairs. Red indicates high interaction probability; blue indicates low. Notable pairs include POSTN–(ITGAV+ITGB5) and GZMA–F2R.

Using AUCell, we quantified ferroptosis activity per cell, identifying malignant cells as the most ferroptosis-enriched population (Mann-Whitney *P < 0.001*, **Figure 10E**). Fibroblasts were stratified into high- and low-ferroptosis groups based on the median value of ferroptosis activity scores. To investigate ferroptosis-mediated intercellular communication, we employed CellChat to infer ligand-receptor interactions across distinct cellular subtypes. The analysis revealed complex multicellular interaction networks involving ferroptosis-associated signaling pathways (**Figures 11A, B**). The POSTN-(ITGAV+ITGB5) ligand-receptor pair exhibited higher communication probability from Lsco_Fibroblasts to Hsco_Fibroblasts, while GZMA-F2R showed elevated interaction likelihood from NK cells to Hsco_Fibroblasts (**Figure 11C**). These results probably implicate fibroblast-mediated crosstalk in ferroptosis-associated TME remodeling.

### Identification of critical model genes in melanoma

**Figure 12:**
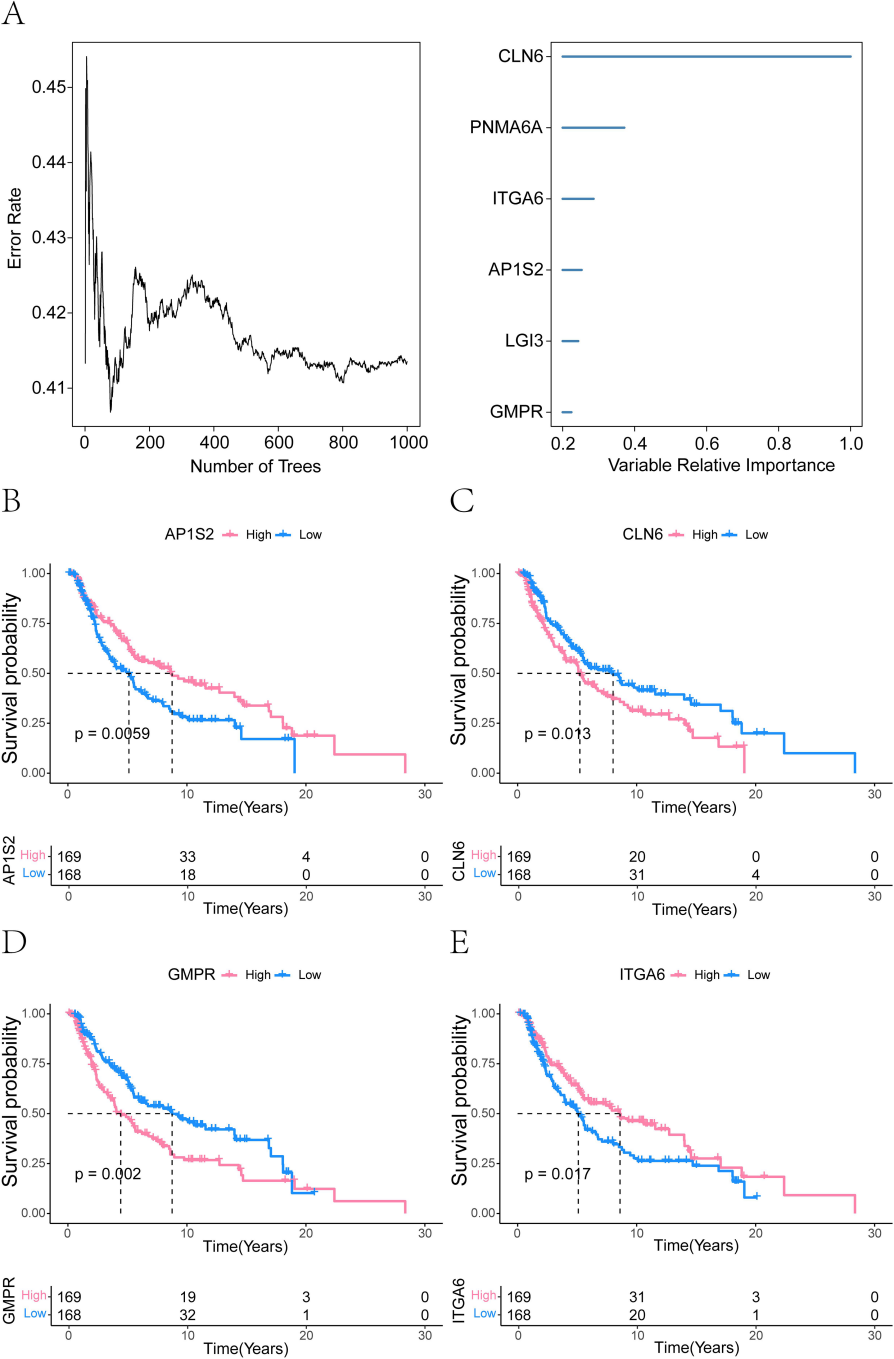
Prognostic model genes associated with melanoma identified by random forest analysis. **(A)** Random forest analysis demonstrating the importance of ferroptosis-related model genes (CLN6, PNMA6A, ITGA6, AP1S2, LGI3, and GMPR). Genes with a relative importance > 0.2. **(B-E)** Survival analysis was performed on the six identified genes, revealing that AP1S2, CLN6, GMPR, and ITGA6 were significantly associated with survival differences (*P ≤ 0.05*).

To further identify key model genes influencing melanoma, we performed RSF analysis on the candidate genes obtained from the aforementioned analyses. Genes with a relative importance > 0.2 were selected as final biomarkers, and the importance ranking of the top 6 genes is displayed in **Figure 12A**. Subsequent survival analysis of these 6 genes revealed that AP1S2, CLN6, GMPR, and ITGA6 exhibited statistically significant differences in survival outcomes (**Figures 12B-E**). Consequently, these four genes were identified as critical model genes for further investigation.

### Validation of prognostic gene expression

**Figure 13:**
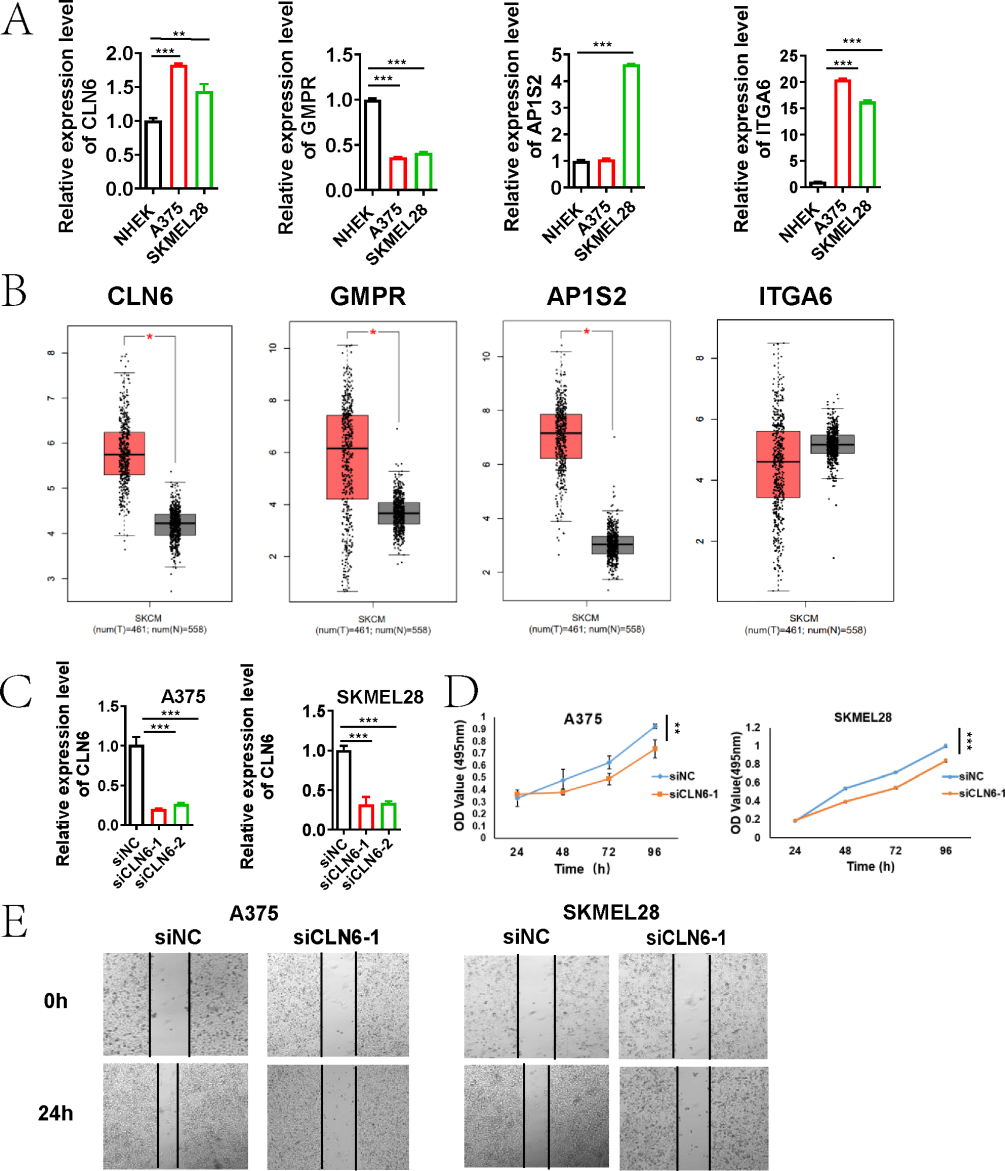
CLN6 as a Critical Driver of Melanoma Proliferation and Migration. **(A)** qRT-PCR validation of the expression of four genes (CLN6, GMPR, AP1S2, and ITGA6) in A375, SK-MEL-28, and NHEK cells. **(B)** Box plots showing the expression of CLN6, GMPR and AP1S2 in 461 skin cutaneous melanoma tissues compared with 558 normal tissues through GEPIA (http://gepia.cancer-pku.cn). **(C)** The knockdown efficiency of CLN6 in A375 and SKMEL28 cells transfected with siRNA1/2 was detected using qRT-PCR. **(D)** Proliferative capacity was detected by MTS assay. **(E)** Wound healing assay was used to detect the migration ability (*P < 0.05, **P < 0.01, ***P < 0.001).

We confirmed the expression patterns of CLN6, GMPR, AP1S2, and ITGA6 in melanoma cells using gene-specific primers by qRT-PCR. CLN6, AP1S2, and ITGA6 were highly expressed in A375 and SK-MEL28 cells, whereas were abundant in HNEK cells (**Figure 13A**). However, we observed low expression of GMPR in A375 and SKMEL28 cells, which contradicted the predicted results. Since all analytical methods may carry inherent limitations or variability, we ultimately excluded GMPR from further investigation.

### CLN6 plays a critical role in melanoma proliferation and migration

Based on GEPIA (http://gepia.cancer-pku.cn), the expression of CLN6, GMPR and AP1S2 was significantly up-regulated in 461 skin cutaneous melanoma tissues compared with 558 normal tissues (**Figure 13B**). Integrated analysis of survival data from the TCGA database, gene expression profiles, and qRT-PCR validation results indicated that CLN6 may serve as a critical prognosis-related gene in melanoma, warranting further investigation and validation. Subsequently, we performed CLN6 knockout and confirmed its efficacy via qRT-PCR (**Figure 13C**). siCLN6–1 was selected for subsequent experiments. In the time-lapse proliferation assay, CLN6 knockdown (siCLN6-1) significantly suppressed cell proliferation at 12, 24, 48 and 96 hours compared to the siNC group (**Figure 13D**). Furthermore, in the scratch wound healing assay, siCLN6–1 knockdown markedly inhibited melanoma cell migration ability (**Figure 13E**).

## Discussion

Melanoma remains the leading cause of skin cancer–related mortality, with more than 833,000 cases reported globally in 2021 (1). Despite advances in targeted therapies (e.g., BRAF/MEK inhibitors) (24, 25) and immunotherapies (e.g., anti-PD-1/PD-L1/CTLA-4) (26, 27), clinical outcomes remain variable, particularly in metastatic disease, where 5-year survival rates drop below 30%. Current staging systems, such as the AJCC TNM classification, are limited by their reliance on clinicopathological parameters, which fail to capture the underlying molecular heterogeneity that drives therapeutic resistance and disease progression (28). To address this unmet need, we developed a ferroptosis-centric risk model that integrates bulk and single-cell transcriptomic data using a machine learning–based framework. This model demonstrated robust prognostic performance, stratifying patients into clinically relevant subgroups with distinct survival outcomes (log-rank *P < 0.0001*) and chemotherapy sensitivities. By linking ferroptosis-associated molecular subtypes with immunological features and clinical phenotypes, our approach contributes to precision oncology by enabling individualized therapeutic decision-making.

The tumor immune microenvironment plays a central role in shaping melanoma progression and therapy response. High infiltration of effector immune cells—such as CD8^+^ cytotoxic T lymphocytes, activated NK cells, and M1 macrophages—has been correlated with improved survival and enhanced sensitivity to immune checkpoint inhibitors (ICIs) (29, 30). In contrast, the increased presence of immunosuppressive populations, including regulatory T cells (Tregs), myeloid-derived suppressor cells (MDSCs), and M2-polarized macrophages, is associated with immune escape and poor prognosis (31–33). Recent advances in single-cell and spatial transcriptomics have revealed substantial intratumoral heterogeneity, including immune “hot” and “cold” phenotypes, that further modulate ICI efficacy (34–37). According to my study, the ferroptosis-immune axis drives melanoma progression through subtype-specific biological mechanisms and immune-metabolic crosstalk. C1 (immune-enriched), characterized by T cell receptor signaling and cytotoxic lymphocyte infiltration (B cells/CD8^+^ T cells), may exhibit a favorable prognosis due to IFN-γ–mediated ACSL4 upregulation, which amplifies ferroptosis sensitivity while maintaining an immune-hot microenvironment responsive to checkpoint inhibitors. In contrast, C3 (lysosomal/epithelial) shows poor survival linked to immunosuppressive Treg/resting mast cell dominance, where TGF-β/IL-10 mediated suppression of cytotoxicity promotes ferroptosis resistance and immune evasion. Paradoxically, lysosomal membrane permeabilization in C3 may transiently enhance iron release via cathepsins, but this vulnerability is overridden by dominant immunosuppression. C2 (mitochondrial/translational) displays intermediate outcomes due to OXPHOS-driven metabolic competition, where nutrient depletion restricts both T cell activity and ferroptosis susceptibility, reflecting metabolic plasticity-driven therapy resistance. These subtypes highlight ferroptosis as a regulatory nexus: immune activation in C1 synergizes with ferroptosis to suppress tumors, while immunosuppression in C3 protects against it. Stromal interactions (e.g., POSTN–ITGB5 signaling) further modulate therapeutic vulnerability, underscoring the need for subtype-tailored strategies-combining immunotherapy/ferroptosis inducers for C1 versus metabolic or stromal targeting for C2/C3.

Previous studies have explored the prognostic relevance of ferroptosis-related genes and long non-coding RNAs (lncRNAs) in melanoma (38, 39). However, most of these models were constructed using univariate selection or limited feature integration strategies. In contrast, our model employed a systems biology approach, incorporating WGCNA-derived hub genes, immune infiltration metrics, and machine learning—based optimization across over 100 algorithms. The resulting 93-gene signature exhibited improved prognostic accuracy and demonstrated predictive relevance for immunotherapy response and chemotherapy sensitivity. Notably, the risk score correlates negatively with cytotoxic T-cell infiltration but positively with immunosuppressive myeloid subsets, mirroring the immune landscape commonly observed in checkpoint-resistant melanomas. Although the StepCox^[both]^ + SuperPC machine learning model achieved a C-index of 0.7 in the training set and 0.61-0.62 in the validation sets, the integration of the genetic risk score with clinical parameters within a prognostic nomogram model (AUC = 0.829-0.845) significantly enhanced the accuracy of prognostic stratification. This improvement underscores the necessity of multidimensional data integration for precision medicine. Future studies shall further validate the potential of the nomogram in guiding immunotherapy and targeted therapy, particularly the potential benefits of personalized interventions for high-risk subgroup patients.

Mechanistically, our data support the dual role of ferroptosis in melanoma—acting both as a tumor-suppressive mechanism and as a modulator of immune escape. While ferroptotic cells can release damage-associated molecular patterns (DAMPs) that enhance antigen presentation, lipid peroxidation byproducts such as 4-hydroxynonenal (4-HNE) may simultaneously impair T cell function and promote Treg survival. Our single-cell analyses revealed that fibroblasts with high ferroptosis activity exhibited increased ligand-receptor interactions with malignant cells, probably through the POSTN–ITGAV/ITGB5 axis. These findings suggest that fibroblast-mediated buffering of oxidative stress may create an immunosuppressive niche that enables tumor progression. Such complexity underscores the need for context-specific targeting of ferroptosis pathways to maximize therapeutic efficacy without compromising antitumor immunity. The four-gene signature (CLN6, GMPR, AP1S2, ITGA6) and their validated roles in proliferation/migration (e.g., CLN6 knockdown suppressing tumor growth) provide novel therapeutic targets. CLN6 is an endoplasmic reticulum (ER) membrane protein, and studies have reported that its functional defects lead to neuronal ceroid lipofuscinosis (NCL), with lysosomal dysfunction being a central hallmark of NCL (40). One key mechanism of ferroptosis involves iron overload-induced lipid peroxidation. As lysosomes are critical organelles for intracellular iron storage and recycling, their dysfunction may disrupt iron metabolism. Additionally, CLN6 deficiency causes impaired lysosomal enzyme trafficking, which could affect the synthesis or localization of GPX4. For example, GPX4 requires transport via the ER-Golgi pathway to reach mitochondria or the cytoplasm, and disrupted trafficking may result in loss of GPX4 function, exacerbating lipid peroxidation (41). A study focusing on glycolysis-related genes and their link to uveal melanoma prognosis identified CLN6 as a gene associated with the prognosis of uveal melanoma (42). In subsequent studies, we will focus on elucidating the mechanistic connections between CLN6 and ferroptosis-related pathways. Additionally, ITGA6 (a laminin receptor) promotes metastasis in multiple cancers, suggesting its inhibition could disrupt ferroptosis-ECM crosstalk. In a study on constructing a prognostic model for glioma based on ferroptosis-related genes, the model gene ITGA6 was verified to enhance cell proliferation, migration, and invasion (43).

Pathway enrichment analyses further elucidated potential mechanisms linking the risk score to disease progression. High-risk patients exhibited signatures of T-cell dysfunction, macrophage immunosuppression (via TIDE), reduced expression of immunotherapy biomarkers (CD274/PD-L1, IFNG), and enrichment in pathways associated with aggressive phenotypes (MYC targets, WNT/β-catenin). Conversely, low-risk scores correlated with enhanced immune responsiveness and sensitivity to specific chemotherapeutics (e.g., Camptothecin, Axitinib) and targeted agents (e.g., Doramapimod). These findings nominate rational combination strategies: for instance, co-administration of MYC inhibitors or WNT inhibitors (e.g., LGK974) with ferroptosis inducers (e.g., erastin) may overcome immune exclusion and sensitize tumors to iron-dependent cell death. On the other hand, the correlation of low - risk scores with enhanced immune responsiveness and sensitivity to certain chemotherapeutics and targeted agents opens up opportunities for personalized treatment strategies. This risk - score - based approach may allow for more precise patient stratification, enabling clinicians to tailor treatments to individual risk profiles and potentially improving clinical outcomes. Further validation in clinical trials is warranted to confirm these findings and pave the way for integrating such risk - score - guided strategies into routine clinical practice.

Several limitations merit consideration. First, this study relied on retrospective analysis of publicly available transcriptomic datasets (TCGA, GEO), and prospective clinical validation is required. Second, single-cell analyses were performed exclusively on primary tumors, potentially limiting generalizability to metastatic lesions with distinct ferroptosis-immune dynamics. Third, the inferred ligand-receptor interactions require experimental confirmation to establish causality. Future work could incorporate spatial transcriptomics to resolve microenvironmental architecture and test ferroptosis-immunotherapy combination regimens in organoid or patient-derived xenograft (PDX) models.

In summary, our study presents an integrative multi-omics framework that decodes melanoma’s ferroptosis–immunity axis. By leveraging bulk and single-cell transcriptomic data, we developed a machine learning—optimized risk model that captures dynamic immunometabolic states and stratifies patients by prognosis, immunotherapy responsiveness, and drug sensitivity. These findings provide a molecular rationale for co-targeting ferroptosis regulators and immune checkpoint pathways to overcome therapeutic resistance in high-risk melanoma. Future translational studies are warranted to validate and extend these observations in clinical settings.

## Data Availability

The datasets presented in this study can be found in online repositories. The names of the repository/repositories and accession number(s) can be found in the article/**Supplementary Material**.

## Supplementary material

Supplementary Image 1 Quality control and preprocessing of single-cell transcriptomic data. **(A)** Violin plots displaying distributions of UMIs, gene counts, and mitochondrial/ribosomal read percentages before and after filtering. **(B)** Scatter plots of mitochondrial vs. nuclear gene expression; doublets (red) removed using Scrublet v2.0.4. Final n = 24,111 cells. **(C)** Identification of top 2,000 highly variable genes (HVGs) via mean–variance plot. **(D)** Log-normalized, z-score scaled expression matrix. **(E, F)** PCA (top 50 PCs) and Harmony-corrected latent space used for batch effect mitigation.

Supplementary Image 2 GO enrichment analysis showing the enriched pathways of the 40 model genes.
