# Supplementary material for: Single-cell and machine learning integration reveals ferroptosis-driven immune landscapes for melanoma stratification
Source: Front Immunol. 2025 Aug 1;16:1624691. doi: 10.3389/fimmu.2025.1624691 (PMC12355379; doi:10.3389/fimmu.2025.1624691)
Supplement: Supplementary file 1 [file Table2.docx]

# **Supplementary Table: Gene Inclusion Overview**

| **Gene Type** | **Model_genes** | **Intersection_genes** |
| --- | --- | --- |
| AFAP1L2 | Yes | Yes |
| AGPAT2 | Yes | Yes |
| AL139246.5 | No | Yes |
| AL162457.2 | No | Yes |
| ALDH3B2 | Yes | Yes |
| ANGPT1 | No | Yes |
| ANKRD9 | Yes | Yes |
| ANXA1 | Yes | Yes |
| AP1S2 | Yes | Yes |
| AVPI1 | No | Yes |
| BAIAP2 | No | Yes |
| BAIAP2L2 | Yes | Yes |
| BBC3 | No | Yes |
| BIRC7 | No | Yes |
| CDH3 | Yes | Yes |
| CDK14 | No | Yes |
| CLN6 | Yes | Yes |
| CYBRD1 | Yes | Yes |
| DIPK1B | No | Yes |
| DIPK1C | No | Yes |
| DLL3 | Yes | Yes |
| ELK3 | Yes | Yes |
| EPS8 | Yes | Yes |
| FAM83H | No | Yes |
| FN3K | No | Yes |
| FNDC10 | No | Yes |
| FZD9 | No | Yes |
| GAS2L3 | No | Yes |
| GDPD5 | Yes | Yes |
| GMPR | Yes | Yes |
| GOLGA7B | No | Yes |
| GPM6B | No | Yes |
| GSTP1 | Yes | Yes |
| GYPC | Yes | Yes |
| HAGHL | No | Yes |
| HES6 | No | Yes |
| HMCN1 | No | Yes |
| HSD17B14 | No | Yes |
| IGSF8 | No | Yes |
| IRX6 | No | Yes |
| ITGA6 | Yes | Yes |
| ITGB3 | Yes | Yes |
| KCNAB2 | No | Yes |
| KCNIP3 | No | Yes |
| KCTD12 | Yes | Yes |
| KU-MEL-3 | No | Yes |
| LGI3 | Yes | Yes |
| MACROD1 | No | Yes |
| MFSD12 | No | Yes |
| MGAT5B | Yes | Yes |
| MMP16 | Yes | Yes |
| MMP17 | No | Yes |
| MSRB2 | No | Yes |
| NAXE | No | Yes |
| NFE2L3 | Yes | Yes |
| NQO1 | No | Yes |
| NT5M | Yes | Yes |
| OCA2 | Yes | Yes |
| OSGIN1 | Yes | Yes |
| PAEP | Yes | Yes |
| PCDH7 | No | Yes |
| PIK3CD-AS2 | No | Yes |
| PLP1 | No | Yes |
| PMAIP1 | Yes | Yes |
| PNMA6A | Yes | Yes |
| RAB3A | No | Yes |
| RAP1GAP | No | Yes |
| REEP6 | No | Yes |
| RENBP | No | Yes |
| RESF1 | No | Yes |
| RHOBTB3 | No | Yes |
| RND3 | No | Yes |
| RPP25 | Yes | Yes |
| RTN4R | Yes | Yes |
| SCARB1 | No | Yes |
| SDC2 | No | Yes |
| SFRP1 | Yes | Yes |
| SHC4 | Yes | Yes |
| SLC27A3 | No | Yes |
| SLC45A2 | Yes | Yes |
| SLC7A4 | Yes | Yes |
| SLC7A8 | No | Yes |
| SLCO4A1-AS1 | No | Yes |
| SPP1 | No | Yes |
| TFAP2A-AS1 | No | Yes |
| TPPP | Yes | Yes |
| TPRN | No | Yes |
| TRIM2 | Yes | Yes |
| TSPAN10 | Yes | Yes |
| TUBB4A | No | Yes |
| UBL3 | No | Yes |
| ZEB1 | No | Yes |
| ZNF703 | Yes | Yes |
