# Supplementary figures and images for: Single-cell and machine learning integration reveals ferroptosis-driven immune landscapes for melanoma stratification
Source: Front Immunol. 2025 Aug 1;16:1624691. doi: 10.3389/fimmu.2025.1624691 (PMC12355379; doi:10.3389/fimmu.2025.1624691)

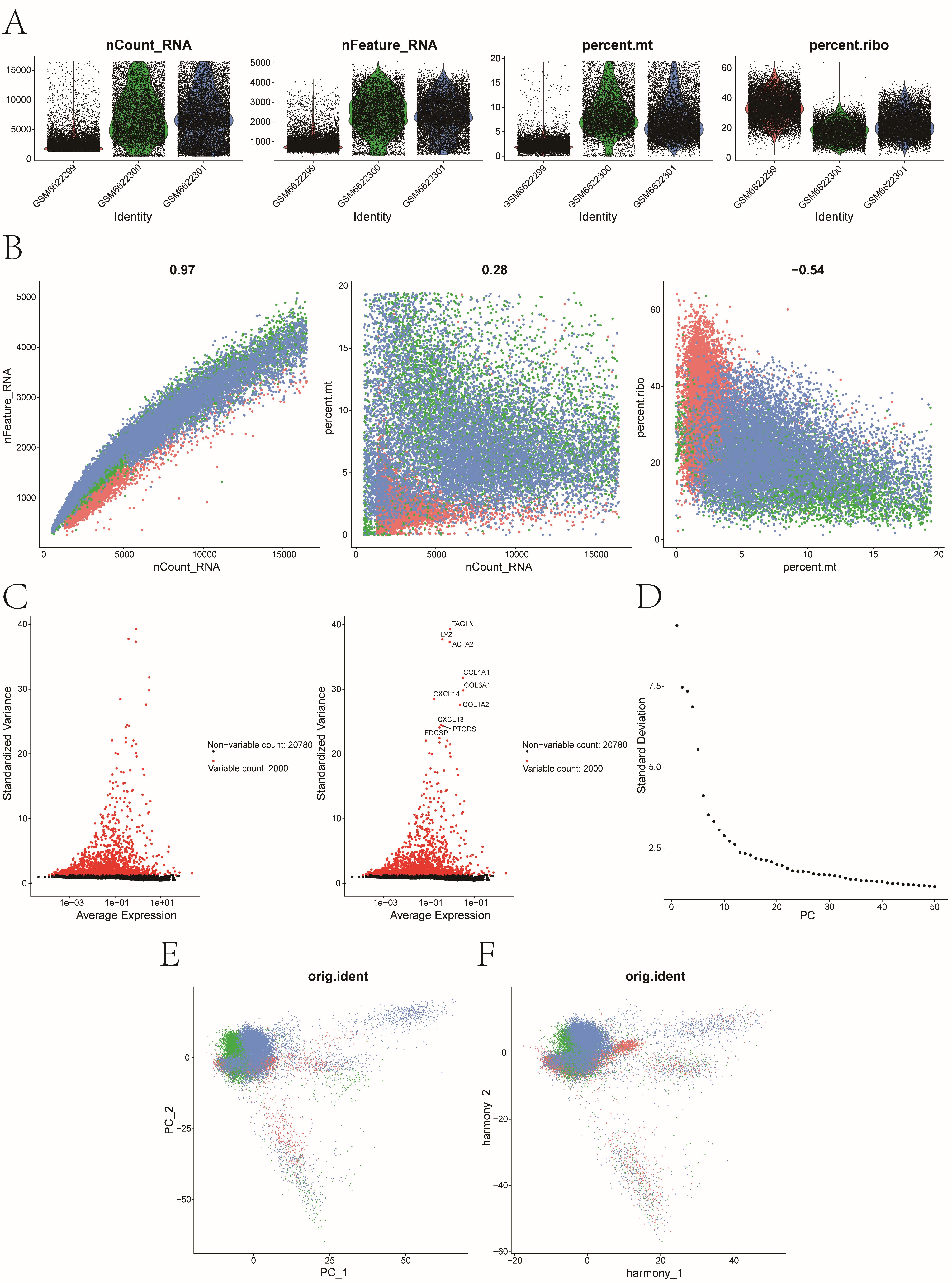

Supplement: Supplementary Image 1 — Quality control and preprocessing of single-cell transcriptomic data. (A) Violin plots displaying distributions of UMIs, gene counts, and mitochondrial/ribosomal read percentages before and after filtering. (B) Scatter plots of mitochondrial vs. nuclear gene expression; doublets (red) removed using Scrublet v2.0.4. Final n = 24,111 cells. (C) Identification of top 2,000 highly variable genes (HVGs) via mean–variance plot. (D) Log-normalized, z-score scaled expression matrix. (E, F) PCA (top 50 PCs) and Harmony-corrected latent space used for batch effect mitigation. [file Image1.jpeg]

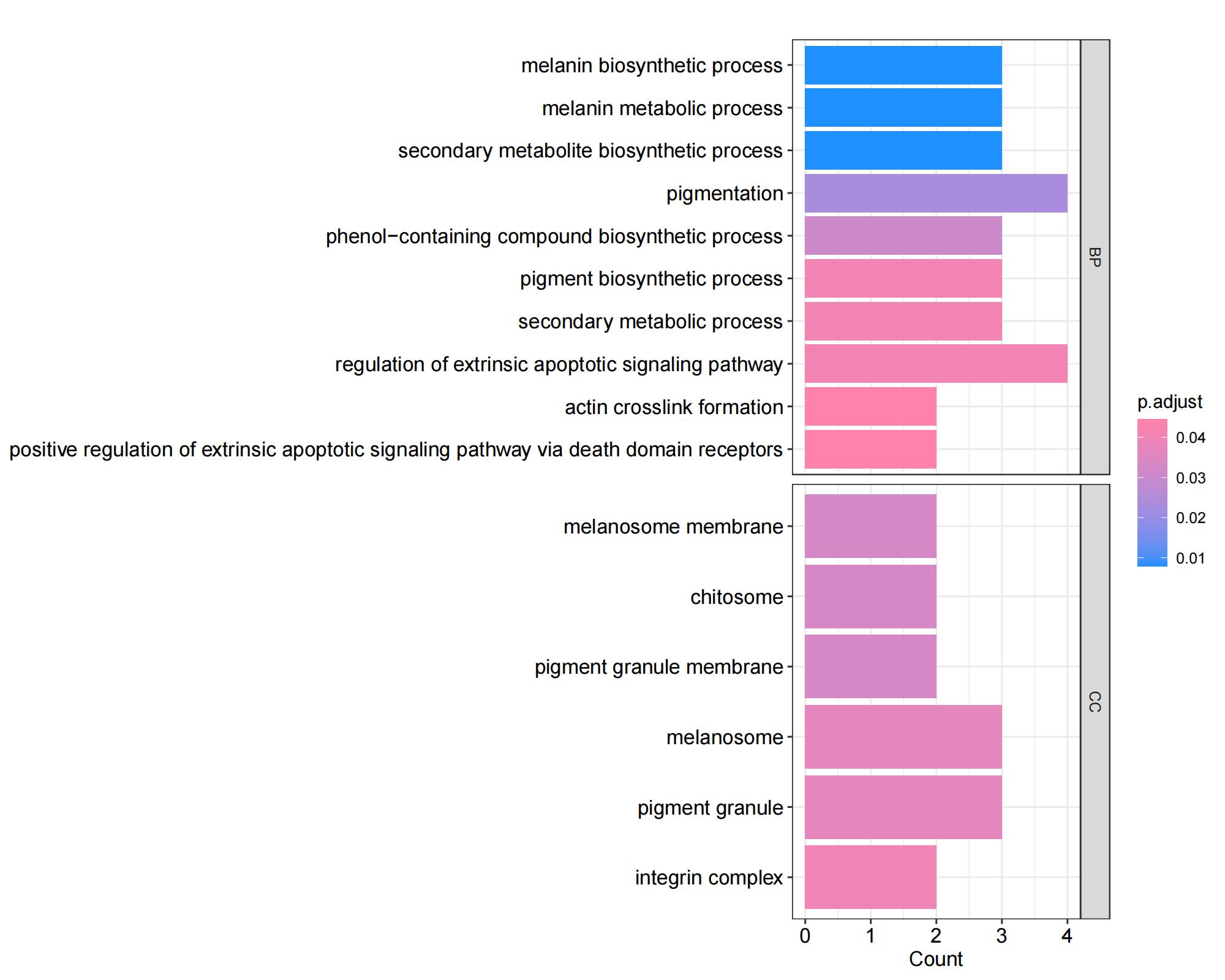

Supplement: Supplementary Image 2 — GO enrichment analysis showing the enriched pathways of the 40 model genes. [file Image2.jpeg]
